# Cognitive Impairment in Strategic Infarct Dementia: A Report of Three Cases

**DOI:** 10.7759/cureus.30009

**Published:** 2022-10-06

**Authors:** Vivek Sanker, Robert Mathew, Maanasi Pranala, Arjun Sudheesh, Vyshnav R Menon

**Affiliations:** 1 General Surgery, Noorul Islam Institute of Medical Science (NIMS), Trivandrum, IND; 2 Neurology, Sree Mookambika Institute of Medical Science, Trivandrum, IND; 3 Surgery, Dr. Somervell Memorial CSI (Church of South India) Medical College, Kerala University of Health Sciences, Trivandrum, IND; 4 Internal Medicine, Indira Gandhi Medical College & Research Institute, Puducherry, IND; 5 Medical School, Washington University of Health and Science, Belize, BLZ

**Keywords:** cerebrovascular accident (stroke), cognitive impairment and dementia, acute infarct, post-stroke dementia, stroke

## Abstract

Strokes involving specific areas regulating cognition and behavioral functions constitute strategic infarct vascular dementia. We present three patients with acute behavioral changes and cognitive impairment following a strategic infarct. Case 1 is of a 59-year-old male, a known patient of diabetes mellitus under treatment, who presented with acute onset of memory deficit along with difficulty in recognizing faces, and left hemispatial neglect. Case 2 is of a 62-year-old male, a smoker, who presented with acute onset of behavioral abnormalities, gait apraxia, and decreased word output. Case 3 is of a 64-year-old female, a known patient of type 2 diabetes mellitus and cerebrovascular accident with left hemiparesis, who presented with psychomotor withdrawal, depression, and cautious gait. One of the most prevalent forms of dementia in adults is vascular dementia, often caused by multiple small strokes, termed multi-infarct dementia. Strategic infarct dementia, on the other hand, is usually caused by a small, single cerebral infarct. The strategic brain regions specifically involved in post-stroke cognitive impairment requires detailed clinical examination along with radiological imaging for accurate localization. Thus the cognitive impact of ischemic strokes can be understood and predicted by clinicians with the help of maps of strategic brain regions associated with global and domain-specific cognitive functions.

## Introduction

Strategic infarct (SI) stroke involves specific areas of the brain critical for cognition and behavior (limbic, associative, paralimbic circuitry) following an ischemic vascular lesion [1]. The most commonly involved sites include caudate nuclei, medial frontal lobe, inferomedial temporal lobe, left angular gyrus, left capsular genu, and thalami [2,3]. The correlation between brain structure and function can be studied from cognitive impairment and the underlying brain damage, making it an interesting entity in clinical neurology. They are mostly associated with acute or gradual onset of vascular cognitive impairment and dementia [4,5].

Patients can present with varying degrees and types of impairment in the different cognitive domains depending on the severity, extent, and location of the lesion. The paramedian nuclei of the thalamus, when involved, can cause impairment in both anterograde and retrograde memory, along with inattention and amotivation [6]. Similarly, when the lesion involves the medial temporal lobe, it causes impairment of episodic memory [7]. We report three cases of cognitive impairment, with varied presentations as a result of focal ischemic lesions of specific brain areas.

## Case presentation

Case 1

A 59-year-old right-handed male, working as a sales executive, presented with acute onset of memory deficit of one-month duration. The patient had a medical history of diabetes mellitus and was under treatment. He found it difficult to learn new things and had problems recollecting recent events. Some events he could recollect with cueing and some of them he couldn’t recollect even with cueing. Memory loss was more for recent events than past events.

**Figure 1:**
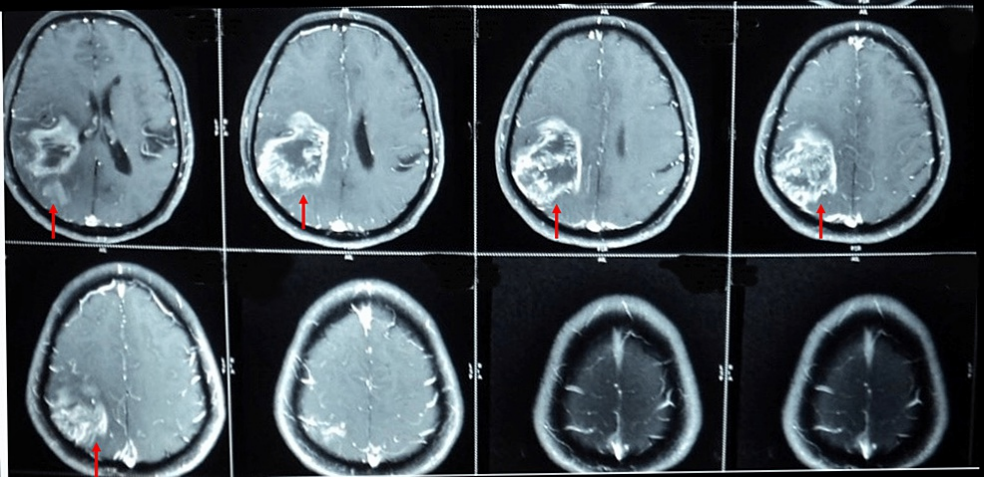
T1W MRI in Case 1 showing relatively well-defined lesion in right parieto-occipital region with peripheral post-contrast enhancement (red arrow). Minimal mass effect to adjacent lateral ventricles and effacement of sulcal spaces; however, no significant midline shift. T1W: T1 weighted

He would often get confused in the middle of a discussion, partly because of memory loss and partly because of word-finding difficulty. He would make mistakes in calculations as in handling money. He also had difficulty identifying familiar places, recognizing known faces, and finding the way back to his home. Left hemispatial neglect was also present. He also found it difficult to locate the sleeve of his shirt, and in buttoning his shirt. On medical evaluation, laboratory test results were within the normal range (complete blood count, electrolytes, thyroid stimulating hormone, viral markers). T1W magnetic resonance imaging (MRI) of the brain showed an ischemic lesion affecting the right parieto-occipital region with hemorrhagic transformation (Figure 1). The patient was treated with long-acting insulin for blood sugar control, anti-platelet drugs, and statin. He made a partial recovery after six months of treatment.

Case 2

A 62-year-old right-handed male presented with acute onset of behavioural abnormalities. He was a smoker for the past 30 years and did not have any other significant medical history. Behavioral abnormalities mostly consisted of disorientation, confusion, and agitation. He had a tendency to forget recent events and at times would confabulate. His word output appeared decreased and would struggle to find the right words. At times he spoke words that did not have real meaning. He talked about irrelevant things at times. He also had incontinence and gait apraxia. He had a history of self fall two months back and sustained a head injury; however, no medical attention had been sought for the same. He was not on any long-term medications.

**Figure 2:**
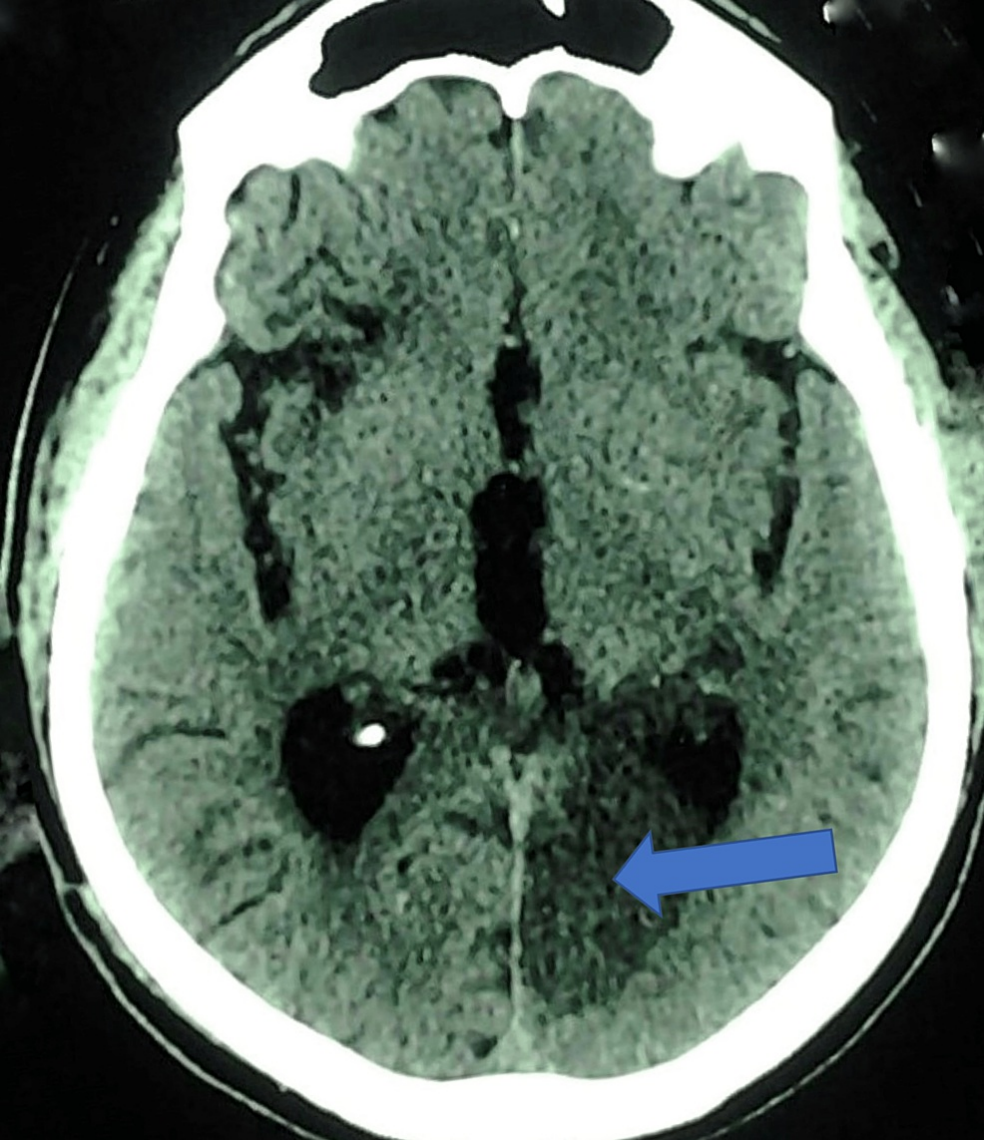
Plain CT axial section in Case 2 showing hypodensity in left medial occipital region with mild mass effect to occipital horn of left lateral ventricle (blue arrow). No hyperdensity within to suggest hemorrhagic transformation; bilateral peri ventricular lucency seen.

His vitals were within normal limits, and systemic examination of the abdomen, respiratory system, and cardiovascular system was normal. The central nervous system (CNS) examination appeared withdrawn, with decreased word output, with no signs of focal deficits, and the fundus showed no papilledema. He was suspected to be a case of SI and computed tomography (CT) scan of the brain showed a focal ischemic lesion involving the left medial occipital area with bilateral periventricular lucency (Figure 2). Following this, medical management was initiated with anti-platelet medications and statins. He underwent physiotherapy at regular intervals and showed a good recovery at four months.

Case 3

A 64-year-old right-handed female, with a history of type 2 diabetes mellitus and CVA with left hemiparesis, presented with psychomotor withdrawal for the past two months. The patient became less active, took an abnormally long time to answer questions, and spoke with a minimal number of words; speech appeared telegraphic. She was reported to take abnormally more time for day-to-day activities like brushing her teeth and taking bath. She also appeared less interested in her surroundings and withdrawn. It appeared that the warmth of interpersonal relationships was lost and she appeared less interested in the emotional well-being of others.

**Figure 3:**
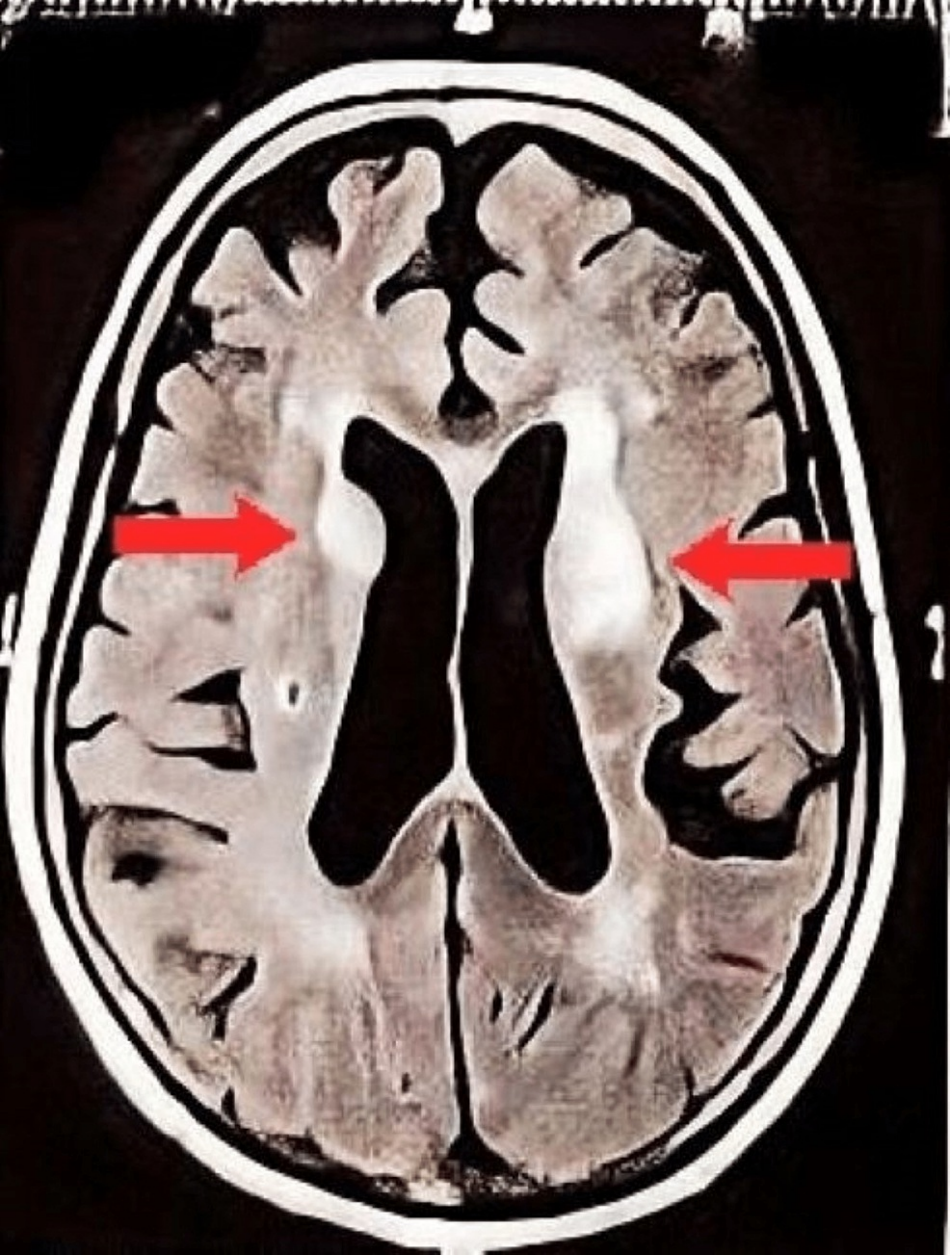
T2W MRI with contrast in Case 3 showing atrophy and microvascular ischemic changes; hyperintensity in bilateral basal ganglia regions suggestive of infarct (red arrow). T2W: T2 weighted

At times she appeared sad and wouldn’t indulge in pleasurable activities. She appeared depressed and with a cautious gait. On examination, higher mental functions seemed affected; she appeared less communicative and depressed. The fundus was not properly visualized. There was no obvious limb weakness but she had a cautious gait. T2W MRI brain showed bilateral basal ganglia infarct, with the involvement of the caudate nucleus, lentiform nucleus, putamen (right), and globus pallidus (Figure 3). She was treated with anti-platelets and statin. The blood sugar was well controlled and, subsequently, she was initiated into speech therapy. She made a good recovery and by the end of three months was almost normal.

## Discussion

One of the most prevalent forms of dementia in adults is vascular dementia, only second to Alzheimer’s disease, and often caused by multiple small strokes or MID [8]. On the other hand, SI dementia is usually caused by a small, single cerebral infarct. Various locations including the caudate nuclei, medial frontal lobe, inferomedial temporal lobe, left angular gyrus, left capsular genu, and thalami are the usual sites of the lesion [9]. They usually present with impairment of cognitive domains (memory, language, attention, executive, general intelligence, or visuospatial) [10]. The cognitive process depends upon several networks executing well-organized coordination and processing between them. A small focal ischemic lesion affecting any of the components of these networks can cause significant impairment and thus has a varied clinical presentation. In all the cases we presented, there were derangements in the above-mentioned domains. The clinical presentation and the degree of impairment depend largely on the site and the extent of the lesion. 

In Case 1, the patient had a lesion of the right parieto-occipital region and presented with left hemispatial neglect due to the involvement of the dorsal stream. He also had an inability to recognize familiar places, faces, and objects due to the involvement of the ventral stream. Parietal areas involved in processing motion and spatial relationships constitute the “dorsal stream,” which receives inputs from distributed networks responsible for visuospatial processing. Hemispatial neglect is manifested by patients having lesions in the right parietal region and the associated networks. Inferior temporal areas involved in processing visual features of a face or an object constitute the “ventral stream” and receive inputs from networks associated with higher-order visual processing [11].

In Case 3, the patient presented with emotional blunting, psychomotor withdrawal, decreased word output, and telegraphic speech. She also seemed to be severely depressed. She had a lesion involving the basal ganglia and the thalamus. She might also have involvement of the medial frontal lobe, which presents as psychomotor withdrawal mimicking features of severe depression [12]. The limbic system comprises the amygdala, thalamus, hypothalamus, and cingulate cortex, and is involved in emotional processing. It has a role in contributing to the consciously experienced emotions as well as in the unconscious functions carried out by the autonomic nervous system. Lesions in the limbic system can have complex behavioral and emotional blunting that often makes the distinction between a neurological and a psychiatric disease difficult.

In Case 2, the patient presented with acute onset of behavioral abnormalities consisting of disorientation, confusion, and agitation. He also had gait apraxia and anterograde amnesia with confabulation. His CT scan revealed a lesion of the left medial occipital area. The symptoms with which the patient presented were indicative of a left posterior cerebral artery (PCA) infarct. The PCA supplies the occipital lobe, inferior part of the temporal lobe, thalamus, midbrain, and posterior limb of the internal capsule. The involvement of the thalamus, hippocampus, and parahippocampus explains his memory loss and confabulation. Patients with PCA stroke can have aggressive behavior as well. They may become anxious, frustrated, and aggressive when they are stimulated by the environment [13].

The ascending reticular activating system arising from the rostral brainstem and projecting to both the thalami and the cerebral hemispheres is associated with arousal. A lesion involving the hemispheres or the rostral brainstem can cause an alteration in arousal and thus the patient can present with altered sensorium, ranging from drowsiness to a vegetative state and coma. The frontal lobe has a major role in attention, which can be assessed by the patient’s ability to avoid distractions and answer directed questions [14]. The dorsolateral prefrontal cortex mainly regulates attention and working memory, which can be assessed by digit span and the patient's ability to spell words backward. Loss of inhibition is seen with lesions of the orbitofrontal cortex.

Broca area in the inferior frontal lobe, arcuate fasciculus, and the Wernicke area in the superior temporal gyrus receive auditory and visual inputs, constituting the language networks in the brain [15]. In the majority of cases, this network is represented in the left hemisphere and with a bilateral or a right hemisphere representation in a smaller proportion of individuals. The various components included in the complete evaluation of language networks include the assessment of writing, reading, comprehension, fluency, repetition, and naming. Aphasia refers to language impairment involving any of the above-mentioned components of the network. Based on the predominant abnormality on examination, it can be conductive, expressive, receptive, or global.

Memory is broadly classified into declarative (episodic memory and recognition memory) and non-declarative (emotional memory, procedural memory, and priming) [16]. The Papez circuit present in the mesial temporal lobe and diencephalon is associated with declarative memory. It also encompasses structures such as the fornix, hippocampus, mammillary body, anterior thalamic nuclei, entorhinal cortex, cingulate cortex, and mammillothalamic tract. Lesions in these areas cause anterograde amnesia, which causes impairment in retrieving recent information. Non-declarative memory, on the other hand, is associated with the striatum, neocortex, amygdala, cerebellum, and reflex pathways.

Various studies have shown the association between the infarct location and the specific cognitive deficit it produces [2,3]. Several strategic regions causing post-stroke cognitive impairment such as corpus callosum, cingulate gyrus, internal capsule, thalamus, basal ganglia, angular gyrus, and frontal subcortical areas have been identified from these studies. The findings from our cases are also in accordance with that of the regions identified from the previous studies of SIs. More specifically, our cases showed the right caudate and lentiform nucleus, and the right and left putamen as strategic regions. Additionally, the right parieto-occipital region and the left medial occipital region were identified as strategic regions for post-stroke cognitive impairment. Our cases can help shed light on the strategic regions, which are likely to be involved in post-stroke cognitive impairment. This information can be vital to clinicians as it can help understand why a lesion in these strategic areas brings about deficits in specific cognitive domains. 

## Conclusions

Our case findings shed light on the strategic brain regions involved in SI that can cause deficits in specific cognitive domains as a result of focal hemorrhage involving the respective region of the brain. Clinicians can predict or understand why a global or domain-specific cognitive deficit occurs following an ischemic lesion of a strategic brain area. Detailed neurophysiological testing and larger sample size can help substantiate the findings of these cases and to develop a model of location-specific infarct and the corresponding cognitive impairment.
